# Highly Sensitive Biosensors Based on Biomolecules and Functional Nanomaterials Depending on the Types of Nanomaterials: A Perspective Review

**DOI:** 10.3390/ma13020299

**Published:** 2020-01-09

**Authors:** Jinho Yoon, Minkyu Shin, Taek Lee, Jeong-Woo Choi

**Affiliations:** 1 Department of Chemical & Biomolecular Engineering, Sogang University, 35 Baekbeom-Ro, Mapo-Gu, Seoul 04107, Korea; moonchild@sogang.ac.kr (J.Y.); mkshin91@sogang.ac.kr (M.S.); 2 Department of Chemistry and Chemical Biology, Rutgers, The State University of New Jersey, Piscataway, NJ 08854, USA; 3 Department of Chemical Engineering, Kwangwoon University, Wolgye-dong, Nowon-gu, Seoul 01899, Korea; tlee@kw.ac.kr

**Keywords:** biosensors, novel nanomaterials, nanoparticles, graphene, transition-metal dichalcogenide (TMD) materials, hybrid nanomaterials

## Abstract

Biosensors are very important for detecting target molecules with high accuracy, selectivity, and signal-to-noise ratio. Biosensors developed using biomolecules such as enzymes or nucleic acids which were used as the probes for detecting the target molecules were studied widely due to their advantages. For example, enzymes can react with certain molecules rapidly and selectively, and nucleic acids can bind to their complementary sequences delicately in nanoscale. In addition, biomolecules can be immobilized and conjugated with other materials by surface modification through the recombination or introduction of chemical linkers. However, these biosensors have some essential limitations because of instability and low signal strength derived from the detector biomolecules. Functional nanomaterials offer a solution to overcome these limitations of biomolecules by hybridization with or replacing the biomolecules. Functional nanomaterials can give advantages for developing biosensors including the increment of electrochemical signals, retention of activity of biomolecules for a long-term period, and extension of investigating tools by using its unique plasmonic and optical properties. Up to now, various nanomaterials were synthesized and reported, from widely used gold nanoparticles to novel nanomaterials that are either carbon-based or transition-metal dichalcogenide (TMD)-based. These nanomaterials were utilized either by themselves or by hybridization with other nanomaterials to develop highly sensitive biosensors. In this review, highly sensitive biosensors developed from excellent novel nanomaterials are discussed through a selective overview of recently reported researches. We also suggest creative breakthroughs for the development of next-generation biosensors using the novel nanomaterials for detecting harmful target molecules with high sensitivity.

## 1. Introduction

Early and accurate detection of harmful or interesting molecules is important from the perspective of the biosensor field to prevent their fatal influence on the body or to cure diseases at an early stage [1,2,3]. To achieve this purpose, biosensors were extensively and intensively studied from the past up until now [4,5]. A biosensor can be defined as an analytical device capable of sensing target molecules such as chemical substances and harmful biomolecules through the specific binding or interaction of these target molecules with sensing materials such as enzymes, antibodies, or designed nucleic acid sequences [6,7,8]. Based on the type of sensing molecule, biosensors can be divided into enzyme-based sensors, DNA-based sensors, immunosensors, and so on [9,10,11]. In addition, various techniques can be introduced for the analysis of biosensing reactions between sensing molecules and target materials, including electrochemical, fluorescence, and optical property analysis, surface plasmon resonance measurements, and surface-enhanced Raman spectroscopy (SERS), which give a chance to develop various types of biosensors dependent on the types of target molecules [12,13,14].

Previously reported biosensors had some essential limitations due to the usage of biomolecules, such as low electrochemical signal strength derived from the biomolecular reaction, instability, and low sensitivity [15,16]. To solve these problems, some materials, including conducting polymers and porous materials, were introduced for immobilization of the biomolecules to increase the electron transfer reaction derived from them and to retain their biomolecular activity [17,18]. Recently, nanomaterials attracted huge attention in a wide range of scientific fields, particularly in the field of biology, because of their properties such as high conductivity and biocompatible cellular uptake [19,20]. The generally known advantages of nanomaterials include extending the activated surface area and creating new features not present in the bulk state [21]. These advantages led to the widespread use of nanomaterials in various fields, from battery electrode research to environmental remediation [22,23,24].

**Figure 1:**
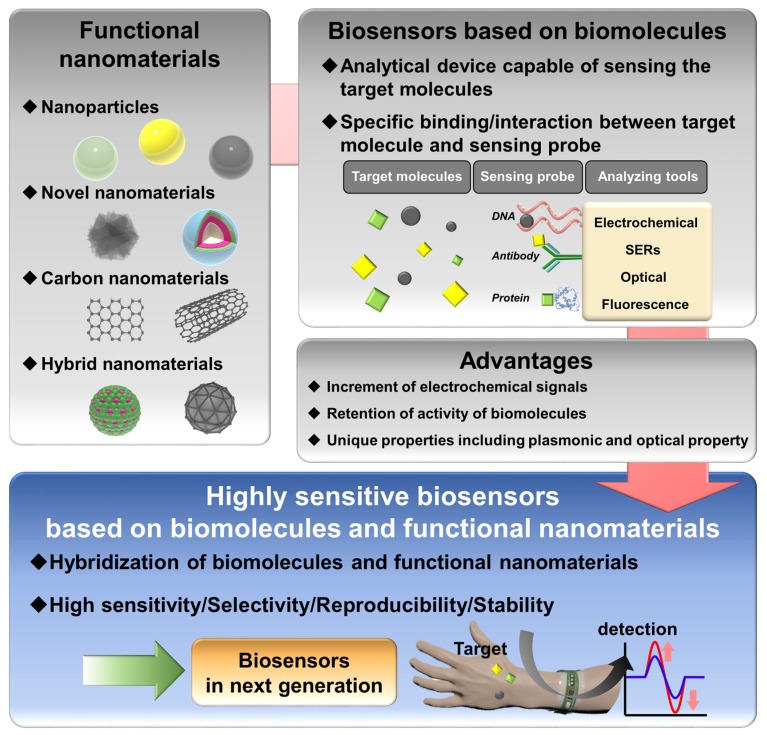
Highly sensitive biosensors based on biomolecules and functional nanomaterials.

In biological fields, the importance of nanomaterials in developing biochips for biological applications such as stem cell therapy and drug delivery increased immensely [25,26], because nanomaterials have advantages for biological application. They can penetrate into the cellular membrane easily because of their nanometer size, and some nanomaterials are biocompatible with cells, making them suitable to be used as the template for delivery of drugs and differentiating inducers directly into the cell and tissue. In addition, magnetic nanoparticles (NPs) covered with biocompatible metal NPs can be exquisitely controlled for delivery of those molecules at the specific location. In particular, in the field of biosensors, nanomaterials signify a new direction for developing highly sensitive biosensors [27]. Through the hybridization of biomolecules and nanomaterials, the advantages of each category can be combined to generate synergetic effects. For example, novel nanomaterials can enhance the SERS signal intensity in SERS-based biosensors, and the low electron transfer signal derived from enzymes can be increased by introducing metal NPs to develop sensitive enzyme-based electrochemical biosensors [28,29]. In addition, the unique optical properties of upconverting NPs (UCNPs) and quantum dots (QDs) are widely used to develop sensitive optical sensors [30]. Furthermore, biosensors in wearable devices are no exception, since recent interest in the latter increased significantly [31]. Recently, flexible biosensors based on polymer substrates were studied extensively, and nanomaterials were introduced to grant conductivity to nonconductive polymers [32,33]. In addition to these examples, various nanomaterials hybridized with biomolecules were widely utilized for the development of highly sensitive biosensors [34,35] (Figure 1).

In this review, highly sensitive biosensors developed by using biomolecules and different types of nanomaterials are discussed in four categories depending on the types of used nanomaterials as NPs, carbon-based nanomaterials, novel nanomaterials, and hybrid nanomaterials. We cover innovative breakthroughs and applications of novel nanomaterials in the development of next-generation biosensors, including practically usable wearable biosensors to detect harmful target molecules with high sensitivity.

## 2. NP-based Biosensors

NPs are the best known and most widely used nanomaterial comprising spherical-shaped structures with a nanometer-sized diameter. Due to their small diameter, NPs possess exceptional properties that do not appear in the bulk product [36,37]. For instance, gold (Au) and silver (Ag) NPs show colorimetric properties depending on the diameter size which can be applied to develop naked-eye colorimetric sensing systems [38]. According to this colorimetric property, a number of lateral flow assay (LFA) biosensors using NPs were reported [39]. For example, an LFA biosensor to detect clenbuterol, a β2 agonist, based on the colorimetric band formation, by using Prussian blue NPs with clenbuterol-specific antibodies, was suggested [40]. Moreover, SERS-based biosensors were developed using the plasmonic properties of specific NPs. As an example, sensitive SERS-based biosensors were reported based on the outstanding SERS activity derived from plasmonic NPs located on the nanogap (1 nm distance between plasmonic NPs) structures on thin SiN membranes for achieving high electric field intensities [41,42]. The plasmonic property of Au nanorods and the magnetic responsive property of Au-coated magnetic NPs were used to develop a bacteria-detecting SERS biosensor through combination with SERS tags and antibodies [43]. In addition to these properties, NPs have other exceptional advantages for developing electrochemical biosensors. It is widely known that metal NPs can enhance the electron transfer reaction of biomolecules and redox molecules [44]. Accordingly, Au and magnetic NPs were used in many electrochemical biosensors, such as using the superlattice structure of Au NPs for amplification of electrochemical signals or using the magnet-based collectable property of magnetic NPs for densely collecting target molecules [45,46]. Some creative researches into recently reported NP-based biosensors are discussed in detail next.

**Figure 2:**
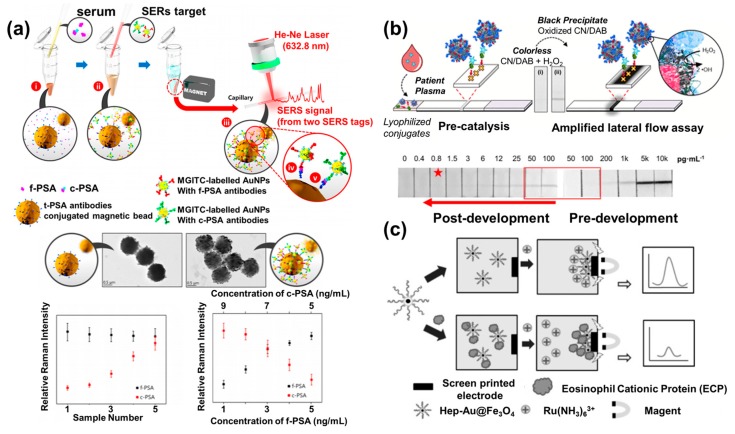
Biosensors developed based on nanoparticles (NPs). (**a**) A schematic of a surface-enhanced Raman spectroscopy (SERS)-based biosensor based on Au and magnetic NPs to detect and distinguish two different prostate-specific antigens (PSAs) for the accurate diagnosis of prostate cancer, with TEM analysis of magnetic NPs with antibodies and formation of a sandwich structure, and SERS results for PSA solutions prepared with different ratio of complexed (c)-PSA and free (f)-PSA (reproduced with permission from Reference [47], published by the American Chemical Society, 2017). (**b**) A highly sensitive optical lateral flow assay (LFA) biosensor based on catalytic Pt NPs for the detection of the human immunodeficiency virus (HIV) p24 biomarker, and detection of colorimetric band formation on the LFA chip before and after addition of hydrogen peroxide to the Pt NPs on the chromogenic substrate (reproduced with permission from Reference [48], published by American Chemical Society (ACS), 2017, (https://doi.org/10.1021/acsnano.7b06229, further permission related to this material should be directed to the ACS)). (**c**) A schematic of an electrochemical eosinophil cationic protein (ECP) biosensor based on Au-coated magnetic NPs (reproduced with permission from Reference [50], published by Elsevier, 2017).

An SERS-based biosensor to detect and distinguish two different prostate-specific antigens (PSAs) using Au NPs, magnetic NPs, and SERS nanotags was developed [47]. To overcome the current limitation for accurate prostate cancer diagnosis, the authors tried detecting the two distinct types of PSA (complexed PSA (c-PSA) and free PSA (f-PSA)) and measured their ratio for more accurate diagnosis of prostate cancer. Detection of only the total PSA amount made it hard to distinguish the prostate cancer from other prostate-related diseases such as benign prostatic hyperplasia and prostatitis, which could induce the increment of total PSA amount. By distinguishing and measuring the ratio of c-PSA and f-PSA, prostate cancer can be predicted accurately through detection of a high ratio of c-PSA compared to f-PSA. To achieve this, they introduced two different SERS nanotags (MGITC for f-PSA and XRITC for c-PSA) conjugated with two distinct specific antibodies for each type of PSA on the Au NPs for targeting the two distinct PSAs. Afterward, magnetic NPs were conjugated with antibodies capable of capturing both c-PSA and f-PSA. Through the sandwich structure formation as shown in Figure 2a, each PSA-targeting hybrid NP was immobilized on the magnetic NPs, and the magnetic NPs with the attached PSA-targeting hybrid NPs were collected using a magnet. Synthesis of magnetic NPs with antibodies and the formation of a sandwich structure were verified by transmission electron microscopy (TEM). From the SERS analysis, the two different PSAs could be detected and distinguished accurately and simultaneously by measurement of the SERS peak intensity of two different SERS nanotags which targeted c-PSA and f-PSA. This biosensing system successfully distinguished the different samples prepared with different ratios of c-PSA and f-PSA (Figure 2a), and the magnet collecting process granted high sensitivity to the proposed biosensor with a sensitive limit of detection (LOD) of 0.012 ng/mL for f-PSA and 0.015 ng/mL for c-PSA. This research provided a manner of developing biosensors, using NPs for precisely collecting and distinguishing the target molecules. As such, the new approach enables developing sensitive biosensors in spite of using NPs developed decades ago.

LFA is one of the most widely studied biosensing platforms because of advantages like low cost and ease of use, which are suitable for point-of-care testing (POCT). However, due to the use of the colorimetric band formed on the LFA for target detection, LFA has a sensitivity problem, as it is tens to hundreds of times lower than the sensitivity of other technique-based biosensors. To increase the sensitivity of LFA biosensors, lots of studies were carried out. Among them, using catalytic platinum (Pt) NPs, a sensitive LFA biosensor was reported for detecting the human immunodeficiency virus (HIV) p24 biomarker (a viral capsid protein of HIV) [48]. Until now, the colorimetric detectable limit value for p24 is below the actual amount of p24 during acute infection. To increase the colorimetric detection limit for an LFA biosensor, the catalytic porous Pt NPs and a chromogenic (CN/DAB (4-chloro-1-naphthol/3,3′-diaminobenzidine, tetrahydrochloride)) substrate located on an LFA chip were introduced in this research. The Pt NPs conjugated with the p24 antibodies were immobilized on the chromogenic substrate of the LFA chip by the formation of a sandwich structure through the capture of p24, as shown in Figure 2b. Through the addition of hydrogen peroxide to the LFA chip, the Pt NPs oxidized the chromogenic substrate, and the apparent colored line could easily be detected visually. Furthermore, the porous structure of Pt NPs could react with hydrogen peroxide efficiently due to the extended surface area which oxidized the chromogenic substrate for colorimetric amplification. This amplification step due to the catalytic properties of the Pt NPs improved the sensitivity of the biosensor significantly, shown in Figure 2b as a 100 pg/mL limit for the pre-development region (before addition of hydrogen peroxide, i.e., the conventional LFA system) and a 0.8 pg/mL limit for the post-development region (after addition of hydrogen peroxide). From the results, the proposed LFA biosensor can detect an extremely low concentration of p24 (0.8 pg/mL) via the colored band on the LFA chip, easily seen with the naked eye. In this way, the proposed biosensor showed a wide detectable range compared with those reported for other biosensors. This demonstrates a new direction for developing highly sensitive LFA biosensors for POCT application.

As mentioned earlier, many electrochemical biosensors based on NPs were reported using the high conductivity and electron transfer facilitating properties of metal NPs. For example, an electrochemical biosensor for detecting microRNA (miRNA) was developed using a uniquely formed bridge DNA aggregated with three Au NPs for amplifying the electrochemical signal through the formation of hugely aggregated Au NP structures and a redox probe for capturing target miRNA [49]. In addition, an electrochemical asthma biomarker-detecting biosensor to detect eosinophil cationic protein (ECP), which is the biomarker for airway diseases such as asthma, in cell cultures was suggested [50]. In this research, the authors used the heparin found on the surface of the cell membrane as the sensing probe that is specifically capable of binding with the ECP. They tried to measure the ECP level in cell cultures using functional magnetic NP modified with heparin. To develop this biosensor, they conjugated heparin with Au-coated magnetic NPs used as the ECP probe with high affinity. Binding of ECP on the heparin-modified Au-coated magnetic NPs induced a positive surface charge, which was otherwise negative in the absence of the ECP. Hexaammineruthenium(III) ions (Ru(NH_3_)_6_^3+^; RuHex) were introduced as the redox generator since they cannot bind with the positively charged ECP-bound heparin-modified Au-coated magnetic NPs because of the positive charge derived from the ECP captured on the functional NPs. However, they can bind with the negatively charged heparin-modified Au-coated magnetic NPs without ECP, as shown in the schematic image of Figure 2c. Fabrication of the biosensor was confirmed by using electrochemical impedance spectroscopy (EIS) and square wave voltammetry (SWV). Depending on the presence or absence of ECP, the redox signals from RuHex were observed when an external magnetic field was applied to the samples collected on the electrode. In this way, the presence of ECP induced a decrease in the electrochemical signal due to the inhibition of the RuHex attachment; a higher concentration of ECP led to a larger decrease in the electrochemical signal by covering the surface of the heparin-modified Au-coated magnetic NPs, as shown in the schematic diagram in Figure 2c. Electrochemical measurements for ECP detection were done by using a CHI 8021 B electrochemical analyzer (CH Instruments) to perform cyclic voltammetry (CV) and amperometric measurements. This biosensor can detect ECP in a wide linear range (1 to 1000 nM) and at an extremely low LOD (0.30 nM) by using the magnetically controllable NPs and the surface charge changes for the detection of target molecules. This research indicates the huge potential for the development of real-time biosensing systems to monitor cellular signals and stem cell differentiation using functional NPs.

In addition to these selected NP-based biosensors, various NPs with unique structures and functions were developed and applied widely in the fabrication of highly sensitive biosensors. Also, mentioned researches provided a new scientific approach to develop sensitive biosensors using widely known NPs which were developed a few decades ago. As such, according to new approaches and perspectives, NPs can be applied not only for biosensors, but also for other scientific fields including batteries and energy fields.

## 3. Carbon-Based Nanomaterials in Biosensors

The application of carbon-based nanomaterials to develop biosensors received huge attention because of their advantages, such as a large surface-to-volume ratio and good chemical stability and biocompatibility [51,52]. For instance, carbon nanotubes (CNTs), carbon nanodots (CNDs), and graphene are the most widely known and used carbon-based nanomaterials [53,54,55], which are suitable for biological application due to their biocompatibility and high conductivity. In particular, CNDs are considered to be candidates for developing fluorescence and electrochemical biosensors due to their unique optical and electrochemical properties [56,57]. For instance, four different types of CND-based sensor arrays were reported for the selective fluorescence detection of several antibiotics such as the tetracyclines, aminoglycosides, amide alcohols, and macrolides by immobilization of different amino acids (isoleucine, leucine, histidine, and tyrosine) on the functional group of CNDs, respectively [58]. In addition, other carbon-based nanomaterials for use in biosensors were reported. Graphene oxide (GO) is a unique material composed of a single layer of graphite with various oxygen-containing functional groups, and it was utilized to develop various biosensors because of its properties such as electrical stability, electrical conductivity, and easy surface functionalization [59,60]. Accordingly, a label-free electrochemical DNA biosensor using reduced (r)GO and conductive polymers was developed to detect a breast cancer gene with high sensitivity and wide dynamic range (1.0 × 10^−14^ to 1.0 × 10^−8^ M). Due to the high conductivity and large surface area of rGO with the conductive polymer, the efficiency for hybridization of target DNA with the single-stranded DNA (ssDNA) probe was increased for effective DNA detection [61].

**Figure 3:**
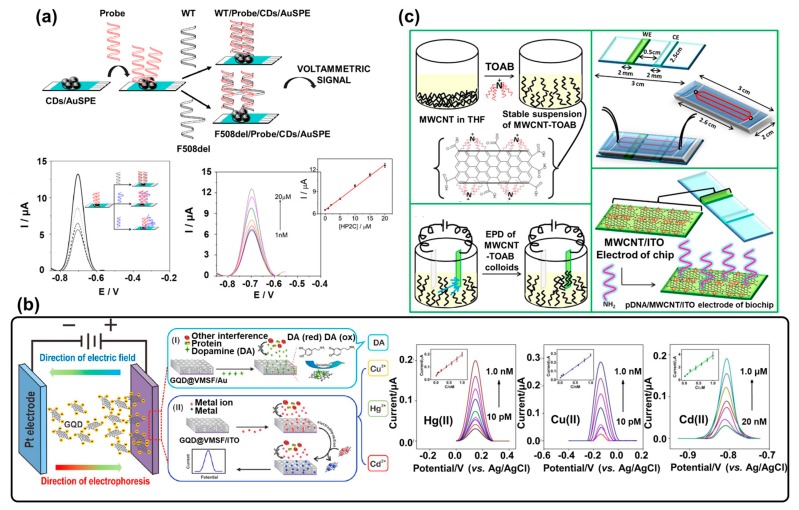
Biosensors based on carbon nanomaterials. (**a**) Schematic images of a DNA biosensor composed of carbon nanodots (CNDs) on a screen-printed disposable electrode to detect gene mutations, and differential pulse voltammetry (DPV) analysis of electrochemical detection performance (reproduced with permission from Reference [62], published by Elsevier, 2018). (**b**) An ultrasensitive electrochemical biosensor based on surface-modified graphene quantum dots (GQDs) to detect metal ions and small molecules, and DPV analysis of Hg(II), Cu(II), and Cd(II) detection (reproduced with permission from Reference [63], published by American Chemical Society, 2018). (**c**) A schematic of an electrochemical microfluidic DNA biosensor based on multiwalled carbon nanotubes (MWCNTs) (reproduced with permission from Reference [64], published by Elsevier, 2018).

Among the fabricated biosensors based on carbon nanomaterials, a DNA biosensor using CNDs synthesized via a thermal carbonation method in a screen-printed disposable electrode was reported [62]. CNDs were composed of a hexagonal lattice of *sp*^2^ carbons incorporated with *sp*^3^ carbons at a size below 10 nm. In addition, these CNDs had numerous functional groups at the surface, which was suitable for easily conjugating with other materials. Conventionally, a DNA biosensor using a traditional sequencing method requires amine-modified oligonucleotides for immobilization onto the carbon-based material via electrostatic or hydrogen bonding. To replace this traditional surface modification of oligonucleotides, CNDs were introduced in the biosensing system, used as a specific nano-sized modifier for the immobilization of unmodified oligonucleotides to detect the target DNA sequences by the formation of hydrogen bonds with the capturing probe DNA bases. In addition, to achieve the rapid and simple detection of specific DNA sequences, safranine (a redox indicator) was introduced into this biosensor as the redox signal generator to detect specific DNA sequences via an electrochemical method. To prepare the biosensor, simple thermal carbonization was initially performed using ethylene glycol bis-(2-aminoethylether)-*N*,*N*,*N*′,*N*′-tetraacetic acid (EGTA) to ensure that the CNDs were effectively immobilized with unmodified oligonucleotides. Next, the synthesized CNDs were immobilized on a prepared screen-printed Au electrode through air-drying, and DNA oligonucleotides as the capturing probe were transferred onto the CNDs immobilized on the screen-printed Au electrode. The electrochemical property of the prepared CND immobilized electrode was confirmed by CV. Due to the effective electron transfer facilitation and extended surface area of CNDs, electrochemical signals derived from safranine, the redox generator, were increased. When the target DNA was hybridized with the capturing probe, the electrochemical signal from the safranine changed depending on the amount of hybridized DNA due to the insulating effect derived from the double-stranded DNA formed between the target DNA and the capturing probe DNA. As shown in Figure 3a, the fabricated DNA biosensor showed that the differential pulse voltammetry (DPV) signal of the fully complementary sequence was increased compared to the single mismatch sequence through the hybridization of the target DNA by the capturing probe. From the results, the current response increased linearly in the 0.001–20 M range, and the detection limit was significantly lower (0.16 nM) when using CNDs compared to without them. This CND-based DNA biosensor is an effective way of detecting gene mutations, and it could be applied to trace the genetic patterns associated with specific diseases.

In another research on carbon material-based biosensors, a graphene (G)QD-based electrochemical biosensor with ultralow detection limits for the measurement of various small analytes in complex samples was developed [63]. To achieve this, GQDs modified with various functional groups and a vertically ordered mesoporous silica nanochannel film (VMSF) were developed and utilized to analyze small analytes in complex samples. Hydroxyl-functionalized GQDs (OH-GQDs) and amine-functionalized GQDs (NH_2_-GQDs) were synthesized using 1,3,6-trinitropyrene as a precursor to detect heavy metal ions such as Hg^2+^, Cu^2+^, and Cd^2+^, which can cause serious health issues and mortality in humans and animals. To fabricate the GQD/VMSF, GQDs were inserted inside nanochannels of VMSF by electrophoresis, after which the electrodeposition of metal ions and the reduction of those ions was performed on the GQD/VMSF for the detection of metal ions. Synthesis of surface-functionalized GQDs was confirmed by TEM, atomic force microscopy (AFM), ultraviolet (UV)–visible light spectroscopy, and X-ray photoelectron spectroscopy (XPS). From the results, the fabricated GQD-based electrochemical biosensor could measure the current peaks of Hg^2+^ and Cu^2+^ via the OH-GQD/VMSF electrode and the Cd^2+^ current peak via the NH_2_-GQD/VMSF electrode. The electrochemical detection was performed using DPV. In addition, due to the surface defects and zig-zag edge structure of the GQD, the electrochemical signal was increased because zig-zag edges were used as the catalyzer for an efficient reaction with metal ions. As shown in Figure 3b, this electrochemical sensor can detect metal ions with good linear correlation in the range of 10 pM to 1.0 nM for both Hg^2+^ and Cu^2+^, and 20.0 nM to 1.0 μM for Cd^2+^ (LODs of 0.3 μM, 32 nM, and 140 nM for Hg^2+^, Cu^2+^, and Cd^2+^, respectively). In particular, dopamine, a small molecule, can be detected efficiently via a fabricated OH-GQD/VMSF electrode through π–π or electrostatic interactions with good linear correlation and an LOD of 120 nM. This fabricated surface-modified GQD/VMSF electrochemical biosensor can suggest a platform which can detect specific biomarkers and small molecules using the unique structure of GQDs.

In addition, an electrochemical microfluidic DNA sensor based on multiwalled (MW)CNTs was developed to detect chronic myelogenous leukemia (CML)-specific DNA [64]. In this research, the conductive material was immobilized uniformly with fast processing time to improve the sensing performance of the electrochemical microfluidic DNA sensor. To fabricate the biosensor, stabilized MWCNTs with tetraoctylammonium bromide (TOAB) were immobilized on an indium tin oxide (ITO) electrode through an electrophoretic deposition method (MWCNTs/ITO), as shown in Figure 3c. Next, the amine-terminated probe DNA was covalently attached to the carboxyl group of the MWCNTs via *N*-ethyl-*N*′-(3-dimethylaminopropyl)carbodiimide (EDC)/*N*-hydroxy succinimide (NHS) to prepare the biosensor. When the probe DNA on the surface of the electrode was hybridized with CML-specific DNA, the charge transfer resistance value of the electrode was changed by immobilization of the target DNA sequences, which induced an increase in the resistance. Fabricated MWCNTs/ITO was confirmed by scanning electron microscopy (SEM) and Fourier-transform infrared spectroscopy (FTIR) spectra, and the detecting performance of the fabricated DNA biosensor was investigated by EIS. Furthermore, the electrical conductivity and electrocatalytic properties of the MWCNTs led to excellent sensitivity in the fabricated electrochemical microfluidic DNA biosensor. The results indicated that the prepared DNA sensor showed enhanced electrochemical detection performance compared with the results obtained by the DNA sensor prepared without MWCNTs. From the results, the fabricated electrochemical microfluidic DNA biosensor can detect targeted oligonucleotides with high sensitivity (1 fM). The MWCNTs provide a large surface area for DNA probe immobilization, and they facilitate electron transfer reactions that result in the highly sensitive detection performance of the biosensor.

As can be seen so far in this section, carbon nanomaterials can provide various advantages, including an extended surface area and high conductivity and biocompatibility, which are suitable properties for developing excellent functional biosensors. In addition, these carbon-based nanomaterials can be used as the template to provide the immobilization location of biomolecules with biocompatibility for biosensing platforms to monitor intracellular signals and in vivo monitoring.

## 4. Novel Nanomaterial-Based Biosensors

Recently, various novel nanomaterials were synthesized and applied in the development of sensitive and stable biosensors. Among them, two-dimensional materials such as transition-metal dichalcogenides (TMDs) and MXenes (few-atom-thick layers of transition metal carbides, nitrides, or carbonitrides) received much attention because of their unique physical, chemical, and electrical properties such as high conductivity, high ion transport efficiency, large activated surface area, and low diffusion barrier [65,66]. Not all TMDs are superior to other nanomaterials including carbon nanomaterials in general. In particular, molybdenum disulfide (MoS_2_), one of the TMDs, is considered to be a more suitable material for the fabrication of biosensors than carbon-based nanomaterials such as graphene due to its excellent electrical properties, a large energy band gap, and semi-conductivity [67]. Using these advantages, high-performance field-effect transistors prepared from chemical vapor deposition (CVD)-grown monolayer MoS_2_ films were used as the platform to detect specific DNA fragments by using the probe DNA immobilized on AuNP-functionalized MoS_2_ [68]. In addition, MXenes were recently applied to develop biosensors because of their advantages of good electrical conductivity, a wide surface area, excellent hydrophilicity, high stability, and biocompatibility [69]. Furthermore, unlike other two-dimensional materials, it is easy to functionalize the groups on the surface of an MXene because functional groups such as OH^−^, O^2−^, and F^−^ are terminated on the MXene during the etching process of the MXene synthesis, which provides an easy way of conjugating biomolecules or other materials on the surface of the MXene. Using these advantages, a mediator-free biosensor composed of Ti_3_C_2_–MXene and hemoglobin to detect nitrite was fabricated. In this research, Ti_3_C_2_–MXene was introduced for an immobilization matrix for redox proteins, with biocompatibility and high conductivity, and hemoglobin was used to detect nitrite via the electrochemical reduction of nitrite [70]. Furthermore, among the various nanomaterials, UCNPs received massive attention because of their optical properties, such as photon upconversion. Generally, UCNPs can convert a short-wavelength (near-infrared (NIR)) input into a long-wavelength (visible or ultraviolet (UV)) output; thus, they can be used in efficient fluorescence probes. In general, UCNPs are prepared by using lanthanide-doped or actinide-doped transition metals, and the emission of high-energy photons through the absorption of low-energy photons was demonstrated to achieve the upconversion mechanism. Furthermore, UCNPs are suitable for blood analysis directly by using NIR wavelength excitation because they can prevent the autofluorescence problem found with most fluorescent dyes [71,72]. For instance, lanthanide-doped UCNPs were developed as a luminescent probe that is effective for bioimaging and biosensing applications due to its low toxicity and high photo-stability. In addition, UCNPs were applied to effective diagnosis of various diseases such as central nervous system diseases and avian influenza virus, as well as direct measurement of the target in blood with high sensitivity and stability without the autofluorescence problem. Accordingly, many fluorescence biosensors using the unique properties of UCNPs were developed, particularly LFA biosensors. As one example, anti-myoglobin-coated UCNPs for the specific quantification of myoglobin (an acute myocardial infarction biomarker) were developed to detect myoglobin by using the fluorescent emitting intensity of anti-myoglobin-coated UCNPs for quantifying the myoglobin amount [73].

**Figure 4:**
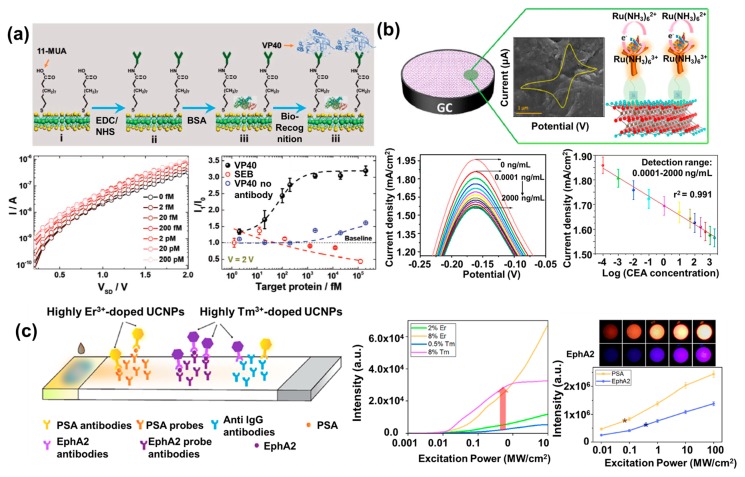
Biosensors developed based on novel nanomaterials. (**a**) Electrochemical biosensor composed of high-quality 2H-MoS_2_ to detect the VP40 matrix protein from the Ebola virus, and I–V characteristics for detecting the antigen–antibody reaction (reproduced with permission from Reference [74], published by John Wiley and Sons, 2019). (**b**) A schematic of the electrochemical biosensor composed of Ti_3_C_2_–MXene (few-atom-thick layer of transition metal carbides, nitrides, or carbonitrides) for sensitive and reproducible carcinoembryonic antigen (CEA) detection, and cyclic voltammogram obtained by using fabricated electrochemical biosensor for detection of different concentrations of target molecules (reproduced with permission from Reference [75], published by Elsevier, 2018). (**c**) A schematic of the LFA biosensor based on highly doped upconverting NPs (UCNPs) to detect PSA and type-A receptor 2 (EphA2) with high sensitivity and selectivity, and photo and fluorescence intensities of testing areas of different concentrations of PSA and EphA2 (reproduced with permission from Reference [77], published by American Chemical Society, 2018).

As an example of TMD-based biosensors, a flexible Ebola virus-detecting biosensor using electrochemically exfoliated high-quality 2H-MoS_2_ flakes was reported [74]. Used here, 2H-MoS_2_ is one type of MoS_2_ depending on the coordination geometry of metal and chalcogen atoms. The structure of the 2H-MoS_2_ featured a Mo atom sitting in a trigonal prismatic environment. To fabricate this flexible biosensor with high sensing performance, 2H-MoS_2_ nanosheets were synthesized using 0.1 M tetra-*n*-butylammonium bisulfate in propylene carbonate via an electrochemical exfoliation method for high quality. To functionalize the 2H-MoS_2_ surface, thiol-containing 11-mercaptoundecanoic acid was anchored to the boundaries or sulfur vacancies of the MoS_2_. Synthesized 2H-MoS_2_ was confirmed by SEM, high-resolution TEM, selected area electron diffraction (SEAD), XPS spectra, and AFM. Afterward, antibodies against the VP40 matrix protein from the Ebola virus were immobilized on the functionalized 2H-MoS_2_ surface via the EDC/NHS reaction. The sensing performance of the fabricated biosensor was confirmed by measuring the current value at 2 V to acquire the I–V (current-voltage) characteristics for detecting the antigen–antibody reaction. When the VP40 matrix protein is bound to the antibodies, the conductivity is changed due to the electrostatic interaction of the biomolecule VP40 matrix protein with the semiconductor material 2H-MoS_2_. In addition, after 100 repeated bending tests with an 8-mm bending radius, the sensing performance of the fabricated biosensor was virtually unchanged. As shown in Figure 4a, the flexible Ebola biosensor showed that the I–V characteristic current signal was increased when the concentration of VP40 matrix protein was increased from 0 fM to 200 pM (lowest detection amount: 2 fM). In addition, this biosensor could detect the VP40 selectively because the signal was detected only when the VP40 antibody was present in the analyte. From the results, the developed flexible biosensor attained a low detection limit of VP40 matrix protein (at the picomolar level) and also had excellent flexibility.

As another example, a titanium carbide (Ti_3_C_2_T_x_)–MXene-based electrochemical biosensor (T_x_ stands for surface termination) to detect the carcinoembryonic antigen (CEA), which is an important cancer biomarker found in lung, breast, and liver cancer patients, was reported [75]. Using the advantages of MXene mentioned before, a multilayered MXene-based biosensor was developed to detect various targets such as nitrite and H_2_O_2_ by combination of MXene with hemoglobin [70,76]. However, the multilayered MXene had limitations of a large surface area and functional group because of the thickness of the MXene layer. From this point of view, single/few-layered Ti_3_C_2_–MXene was synthesized by etching the aluminum (Al) layer in Ti_3_AlC_2_ using lithium fluoride (LiF) solution and HCl. Next, the surface of the synthesized Ti_3_C_2_–MXene was functionalized with amine groups using (3-aminopropyl) triethoxysilane (APTES) for CEA-antibody immobilization. Subsequently, the surface-modified Ti_3_C_2_–MXene was immobilized on a glassy carbon electrode by drop-casting and drying at room temperature. Synthesis of the surface functionalized Ti_3_C_2_–MXene was verified by XRD pattern, TEM, elemental mapping, and XPS spectra. The electrochemical sensing performance of fabricated biosensor was investigated by CV using hexaammineruthenium ([Ru(NH_3_)_6_]^3+^) as the redox probe. In general, the widely used [Fe(CN)6]^−3/−4^ as a redox probe has a problem of oxidation of Ti_3_C_2_–MXene in the measurement range. To prevent this problem, [Ru(NH_3_)_6_]^3+^ was used as the redox probe in this research. As shown in Figure 4b, when the specific antigen was bound to the CAE antibody, the electrochemical signal of the biosensor was reduced due to a decrease in the charge transfer. The biosensor showed good linear correlation in the wide detection range of 0.001–2000 ng/mL with good sensitivity. In addition, it retained its biosensing property for seven days with high reproducibility. This research suggested the possibility of using Ti_3_C_2_–MXene in biosensor applications.

As mentioned before, UCNP-based biosensors that exploit their fluorescent property were reported extensively, particularly in LFA biosensors. In general, large-sized UCNPs up to 400 nm with lots of emitters were used to induce the high brightness of emitted signal from UCNPs. However, large-sized UCNPs hindered the effective flow of UCNPs through the porous region of the LFA chip composed of a nitrocellulose film to develop the biosensors. To solve the size-related problem, small-sized UCNPs (<100 nm) were synthesized and modified with densely doped emitters to synthesize UCNPs capable of efficiently emitting high luminescence with a small size. Using the small-sized UCNPs with densely doped emitters, LFA biosensors were developed to detect PSA and type-A receptor 2 (EphA2). To detect target molecules, the sensing probes were combined with UCNPs which were a prostate cancer biomarker and pancreatic cancer biomarker [77]. To develop this biosensor, highly doped UCNPs composed of erbium (Er^3+^) and thulium (Tm^3+^) ions were synthesized to achieve an excellent brightness emission signal and sensitivity. Syntheses of two kinds of highly doped UCNPs were confirmed by TEM, power-dependent spectra, and dynamic light scattering. UCNPs were conjugated with anti-PSA and anti-EphA2 antibodies to detect PSA and EphA2. When the target analyte is bound to the antibodies immobilized on the highly doped UCNPs, it can be detected by the increase in emission intensity from the LFA biosensor. As shown in Figure 4c, the fabricated LFA biosensor exhibited emitted fluorescence intensity that was five times higher for Er^3+^ and 12 times higher for Tm^3+^ compared to the intensity from the lower-doped UCNPs. The proposed UCNP-based LFA biosensor can detect target analytes with ultra-sensitivity, selectivity (LODs of 89 pg/mL for PSA and 400 pg/mL for EphA2), and good linear correlation (0.01 ng/mL to 100 ng/mL). Also, the target detection was performed with a strip test system based on a single-photon avalanche detector (SPAD) through colorimetric change detection by using the camera of a cellular phone. This UCNP-based LFA biosensing system can be applied to develop portable and wearable devices for the detection of a wide range of biomarkers and analytes.

As discussed herein, there were various recently developed novel nanomaterials for application in the biosensor field, and the process is ongoing. In addition, reported novel nanomaterial-based biosensors showed excellent biosensing performances, and they were widely utilized in various biosensing tools by applying various electrochemical and fluorescence methods, thereby showing their versatility. Furthermore, this nanomaterials-based detection of target molecules can be easily achieved using cellular phones.

## 5. Hybrid Nanomaterial-based Biosensors

In addition to the various nanomaterials mentioned earlier, hybridization of these nanomaterials with other materials was utilized to exploit their exceptional functionality and to develop outstanding hybrid nanomaterials for application in various scientific fields [78,79,80]. The developed hybrid nanomaterials showed excellent electrochemical, plasmonic, and photoluminescent properties not present before the hybridization [81,82]. For example, one plasmonic hybrid nanomaterial developed via a combination of NPs and semiconducting materials simultaneously showed two different charge transfer mechanisms, which could provide a new perspective for photoinduced carrier dynamics and could be applied for photocatalysis or photovoltaics [81]. Furthermore, many of the developed hybrid nanomaterials were used to develop sensitive and functional biosensors [83,84]. Among these, Choi et al. fabricated a GO and MoS_2_ hybrid nanomaterial and utilized its exceptional electron transfer property to increase the redox signal derived from metalloprotein to sensitively detect hydrogen peroxide [85]. As another example, cobalt oxide (Co_3_O_4_) NPs and lead sulfide (PbS) NPs were decorated on an ITO photoelectrode to develop a photoelectrochemical hydrogen peroxide biosensor using the photoelectrochemical property of PbS and multienzyme activity derived from Co_3_O_4_ [86]. In addition, an intracellular dual miRNA biosensor fabricated using hybrid nanopyramids composed of Au–copper sulfide (Cu_9_S_5_) NPs, UCNPs, and silver sulfide (Ag_2_S) NPs, which can emit two luminescent signals simultaneously for dual miRNA detection, was reported [87].

**Figure 5:**
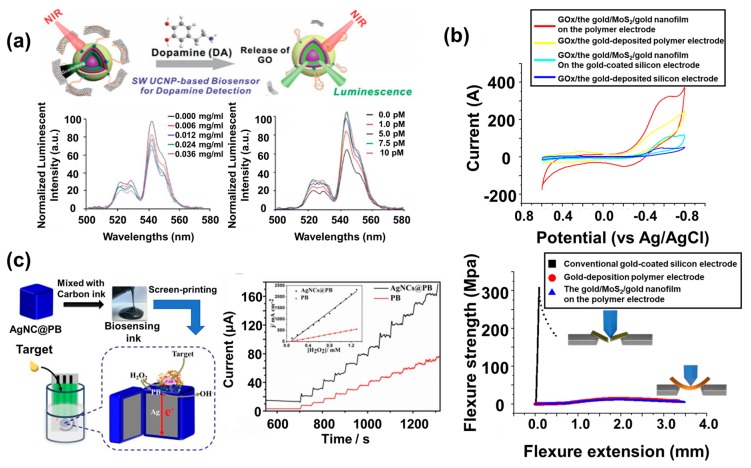
Biosensors based on hybrid NPs. (**a**) A dopamine biosensor composed of graphene oxide (GO)-encapsulated sandwich-structured UCNPs, and its sensitive dopamine detection performance obtained by fluorescence spectrophotometer (reproduced with permission from Reference [88], published by John Wiley and Sons, 2019). (**b**) Cyclic voltammograms of a flexible electrochemical biosensor based on an Au/MoS_2_/Au hybrid nanofilm on a polyimide (PI), and its ability for flexibility acquired by using a fatigue tester (reproduced with permission from Reference [32], published by Elsevier, 2019). (**c**) Schematic images of flexible biosensing platform based on silver nanocubes (NCs) encapsulated by Prussian blue (AgNCs@PB) bio-ink, and the amperometric response following the addition of hydrogen peroxide (reproduced with permission from reference [96], published by John Wiley and Sons, 2016).

Among the various hybrid nanomaterials used in biosensors, an interesting one was developed and utilized in a biosensor for detecting neurotransmitters produced by neurons [88] (Figure 5a). A sandwich structure of UCNPs composed of an ytterbium (Yb)-doped core, an Er-doped luminescent shell, and a Yb-doped sensitizing shell to achieve enhanced upconversion luminescence was synthesized and encapsulated with GO using an aptamer capable of specifically binding with dopamine. The structure of proposed UCNPs enabled energy back-transfer mitigation for increased upconversion luminescence emission. When the dopamine binds to the aptamer of the developed hybrid nanomaterial as shown in Figure 5a, the structural change in the aptamer by connecting with the dopamine induces the release of GO from the hybrid nanomaterials, and the enhanced luminescence signal from the hybrid nanomaterial can be detected due to the release of GO, which blocks the luminescence emitted by the UCNPs activated by NIR laser exposure. In this mechanism, the dopamine from the differentiated dopaminergic neurons was successfully detected by measuring the luminescence intensity using a fluorescence spectrophotometer with external NIR lasers and an optical power meter with an 818P thermophile detector. Synthesis of sandwich structural UCNPs was verified by TEM, scanning transmission electron microscopy (STEM), and electron energy loss spectroscopy (EELS). As shown in Figure 5a, this biosensor could detect the 1 pM amount of dopamine by luminescence increment due to the detection of dopamine, which was much more sensitive compared to the recently reported dopamine biosensors developed based on conventionally used Ag NPs (detection limit: 0.6 μM) [89]. In addition, by hybridization of UCNPs and GO, which was used for blocking the emission of luminescence, the proposed biosensing system showed a novel method for increasing the sensitivity for dopamine detection, which was much more sensitive than the biosensor developed only using UCNPs (detection limit: 1.62 μM) [90]. Furthermore, the newly synthesized sandwich-structured UCNPs showed a significantly enhanced upconversion luminescence signal by NIR excitation compared to other reported UCNPs. The results of this research suggest a creative direction for developing functional novel hybrid nanomaterials using the essential property of each component.

Recently, flexible biosensors attracted much attention due to the increased requirement for wearable healthcare devices [33,91]. Electrochemical techniques are suitable for developing wearable biosensing devices because of their advantages including ease of miniaturization, fast/sensitive response, simplicity, and low energy requirement [92]. Flexible polymer materials such as polyimide (PI) and poly (ethylene terephthalate) (PET) are widely used as the substrate in biosensors. However, the nonconductive property of those polymers needs to be overcome for use in electrochemical flexible biosensors. A number of techniques, such as photolithography, printing, chemical vapor deposition, and electron-beam evaporation, were used to form a conductive layer on the polymer substrate to grant conductivity to the device [93,94,95]. Among these techniques, Choi et al. [32] suggested a simple and easy method to fabricate an electrochemical flexible biosensor on a PI substrate. They spin-coated MoS_2_ NPs and deposited Au ions by sputter deposition onto a PI substrate to fabricate an Au/MoS_2_/Au hybrid nanofilm with excellent electron transfer characteristics for enhancing the redox reaction between glucose oxidase and the glucose. High blood glucose is an important bioindicator of diabetes mellitus (a disease that can induce disability and death in humans). Glucose oxidase, the sensing metalloprotein which has a metal ion in its core that reacts with glucose electrochemically, was immobilized on the fabricated hybrid nanofilm through the chemical linker, 6-mercaptohexanoic acid (6-MHA). Fabrication of the Au/MoS_2_/Au hybrid nanofilm was confirmed by SEM and energy-dispersive X-ray spectroscopy (EDX) mapping. Immobilization of glucose oxidase was verified by CV through the detection of redox signals derived from immobilized glucose oxidase. As shown in Figure 5b, the prepared biosensor showed an enhanced redox signal derived from the glucose oxidase (a reduction peak current value of 323.0 μA) compared to the control due to the introduction of the Au/MoS_2_/Au hybrid nanofilm on the polymer substrate to facilitate the electron transfer. By using the amperometric I–T (current-time) technique, the biosensor could detect glucose excellently (a 10 nM detection limit) and selectively in human serum through the detection of generated electrons by electrochemical reaction between glucose oxidase and added glucose. In addition, it retained its conductive property without the hybrid nanofilm cracking even after numerous bending tests, and its flexibility was verified by using the micro fatigue tester. The most important thing in this research is that the flexible conductive substrate composed of hybrid nanomaterial layers was developed via a simple fabrication process that can be easily applied to the development of other biosensing platforms.

In addition to the aforementioned flexible biosensor, a hybrid nanomaterial composed of Ag nanocubes (NCs) encapsulated by Prussian blue (PB) (AgNCs@PB) was synthesized and used as the template to develop a flexible biosensing platform for detection of hydrogen peroxide [96]. Based on the conductive property of AgNCs and electrocatalytic property of PB, authors synthesized the core–shell structural AgNCs@PB to integrate the properties of each nanomaterial. In general, PB is treated as an artificial peroxide because it can react with hydrogen peroxide. However, it is included in the semiconductor; thus, it hinders the effective electron transfer reaction. To overcome this limitation, AgNCs were introduced to promote the electron transfer. AgNCs could be tightly encapsulated by PB through the electrostatic attraction between the negative charge of PB and positive charge of AgNCs. Furthermore, synthesized AgNCs@PB had proper viscosity by mixing with carbon ink, which could be used as the bio-ink for screen printing to fabricate the biosensor. Using the 245 DEK screen printing machine, AgNCs@PB bio-ink was printed on the flexible PET substrate to fabricate the working electrode. In addition, counter and reference electrodes were also fabricated by printing of carbon ink and silver chloride ink on the PET substrate. Synthesis of AgNCs@PB was confirmed by TEM, SEM, selected area electron diffraction (SAED) analysis, XRD, and XPS. By EDX mapping analysis, fabrication of the screen-printed electrodes was verified. The developed flexible biosensor showed the enhanced redox property derived from PB due to the existence of AgNCs compared to the pure PB. In addition, this biosensor could sensitively detect hydrogen peroxide (LOD: 2 μM) by amperometric investigation, which (calibrated sensitivity: 1822.96 μA∙mM^−1^∙cm^−2^) was four times higher than the result of the biosensor prepared with only PB (calibrated sensitivity: 450.55 μA∙mM^−1^∙cm^−2^) (Figure 5c). Including the high sensitivity, this flexible biosensing system could suggest a facile and large-scale flexible biosensor fabrication strategy using hybrid nanomaterials.

Including the previously discussed researches, hybrid nanomaterials can be utilized to develop highly sensitive biosensors and flexible electronic devices applied not only in sensors but also in wireless devices. In addition to wide applications in the field of biosensors, novel hybrid nanomaterials can be used widely in biological fields such as cell fate monitoring, spatiotemporal release of drugs, and regenerative medicine. Furthermore, due to its exceptional electrical, electrochemical, and optical properties, developed hybrid nanomaterials can be utilized in various scientific fields such as electrodes for batteries and energy generation [97,98].

## 6. Conclusions and Future Perspective

Since the development of nanotechnology began in earnest, various novel nanomaterials were developed and applied in numerous scientific fields. Those nanomaterials provided huge potential in biological fields, particularly in the field of biosensors, because of their superior properties, including electron transfer facilitation, unique optical and plasmonic effects, and biocompatibility, compared with their bulk counterparts. In addition, the exceptionally outstanding properties of nanomaterials helped to solve the intrinsic limitations of biomolecules such as instability and extremely low signals derived from biomolecular reactions, as well as grant high sensitivity to biosensors using their properties such as the high conductivity, efficient fluorescence emission, and reacting properties with harmful molecules. Accordingly, the advantages produced by the combination of biomolecules and nanomaterials are useful for achieving the ultimate goal in the field of biosensors, which is the detection of harmful and interesting molecules with high sensitivity and selectivity.

Accordingly, numerous superb biosensors were developed using biomolecules and nanomaterials. In this review, we discussed biosensors developed by using biomolecules and various types of nanomaterials. The discussion was conducted sequentially according to four categories of different types of nanomaterials. Firstly, we investigated biosensors based on NPs, the most widely known type of nanomaterial, used in SERS-based, LFA-based, and electrochemical biosensors, and these researches suggested a new approach for developing biosensors using NPs. Next, we discussed biosensors based on carbon nanomaterials, which attracted much attention in recent years. In addition, we considered biosensors developed by using nanomaterials with advantages including luminescence and electrochemical properties, such as UCNPs and MoS_2_. Finally, we discussed biosensors constituting biomolecules and hybrid nanomaterials with two or sometimes more different novel nanomaterials, including UCNPs with graphene, and AgNCs encapsulated by PB. Those hybrid nanomaterials showed synergetic effects via the conjugation of two different nanomaterials for developing highly sensitive biosensors. Furthermore, these hybrid nanomaterials provide the possibility for the development of wearable biosensors, which is currently attracting huge attention. Table 1 shows the functional nanomaterials discussed in this review in detail. In conclusion, the original researches covered in this review provide new approaches and inventive breakthroughs for developing next-generation biosensors, with a wide scientific application of nanomaterials.

## Figures and Tables

**Table 1:** Comparison and characteristics of nanomaterials utilized in highly sensitive biosensors.

Target	System	Nanomaterial	Sensing Probe	Advantage	Detection Limit	Ref.
f-PSA c-PSA	PSA/MGITC@AuNPs/magnetic NPs	AuNPs/magnetic NPs	f-PSA antibody, c-PSA antibody	Effective collection of probe NPs for enhancing the sensitivity	0.012 ng/mL (f-PSA), 0.15 ng/mL (c-PSA)	[47]
HIV p24	Ab-PtNPs/HIV p24/Nanobody-biotin/polystrept abidin	PtNPs	HIV p24 antibody/PtNPs conjugates	Apparent colorimetric band formation by catalytic reaction derived from porous PtNPs	0.8 pg/mL	[48]
eosinophil cationic protein (ECP)	Hep-Au@Fe_3_O_4_	Au@Fe_3_O_4_	Hep-Au@Fe_3_O_4_	Effective collection of probe NPs for enhancing the sensitivity	0.3 nM	[50]
DNA	DNA/Probe/CDs/AuSPE	CDs	Unmodified oligonucleotides	Synthesis of surface modified CDs immobilized with unmodified oligonucleotides	0.16 nM (100 times higher than sensor prepared without CDs)	[62]
DA, Hg^2+^, Cu^2+^, Cd^2+^	GQDs/VMSF	GQDs	OH-GQDs, NH_2_-GQDs	Effective detection of various type of metal ion and small molecule using surface modified GQDs	120 nM (DA) 0.3 μM (Hg^2+^) 32 nM (Cu^2+^) 140 nM (Cd^2+^) (3 times higher than sensor prepared without GQDs)	[63]
Nucleic acid	pDNA/MWCNT/ITO	MWCNT	Amine terminated DNA	Electrochemical signal enhancement	1 fM (10,000 times higher than sensor prepared without MWCNT)	[64]
VP40	VP40/BSA/MoS_2_/Au electrode	MoS_2_	VP40 antibody	Electrochemical signal enhancement	2 fM	[74]
Carcinoembrynic antigen (CEA)	Anti-CEA/*f*-Ti_3_C_2_/GC	Ti_3_C_2_	Carcino embryonic antibody monoclonal	Synthesis of surface modified Ti_3_C_2_ and electrochemical signal enhancement	0.018 pg/mL	[75]
PSA, EphA2	Highly doped UCNPs	UCNPs	PSA antibody, EphA2 antibody	Luminescence signal enhancement using highly lanthanide ion doped UCNPs	89 pg/mL (PSA), 400 pg/mL (EphA2) (5 times and 12 times higher than lower doped UCNPs)	[77]
DA	GO/Aptamer/SW-UCNPs	GO/UCNPs	DA-specific aptamer	Luminescence signal enhancement due to the sandwich structure of UCNPs	~1 pM	[88]
glucose	GOx/Au/MoS_2_/Au/PI	Au/MoS2/Au nanofilm	Glucose oxidase	Electrochemical signal enhancement	10 nM (3 times higher than sensor prepared without MoS_2_)	[32]
H_2_O_2_	AgNCs/PB/PET	AgNCs	Glucose oxidase	Electrochemical signal enhancement	2 μM (4 times higher than sensor prepared without AgNCs)	[96]
